# Innovative Therapeutic Approaches Targeting Obesity: Can Flavonoids Improve the Efficacy of Anti-Obesogenic Drugs?

**DOI:** 10.3390/ijms262010142

**Published:** 2025-10-18

**Authors:** Emanuele-Salvatore Scarpa, Stefano Amatori, Giovanni Caprioli, Filippo Maggi, Gianluca Moroncini, Giancarlo Balercia, Gilberta Giacchetti

**Affiliations:** 1 R&D Department, Mivell S.r.l.s., 61032 Fano, Italy; 2 Department of Biomolecular Sciences, University of Urbino Carlo Bo, 61032 Fano, Italy; stefano.amatori@uniurb.it; 3 School of Pharmacy, Chemistry Interdisciplinary Project (ChIP) Research Center, University of Camerino, Via Madonna delle Carceri, 62032 Camerino, Italy; giovanni.caprioli@unicam.it (G.C.); filippo.maggi@unicam.it (F.M.); 4 Department of Clinical and Molecular Sciences, Università Politecnica delle Marche, 60126 Ancona, Italy; g.moroncini@staff.univpm.it; 5 Division of Endocrinology, Department of Clinical and Molecular Sciences, Università Politecnica delle Marche, 60126 Ancona, Italy; giancarlo.balercia@ospedaliriuniti.marche.it; 6 Clinic of Endocrinology and Metabolic Diseases, Department of Clinical and Molecular Sciences, Università Politecnica delle Marche, 60126 Ancona, Italy

**Keywords:** obesity, flavonoids, anti-obesity drugs, anti-adipogenic, anti-obesogenic, anti-inflammatory, antioxidant

## Abstract

Obesity is a chronic, multifactorial metabolic disease associated with various factors such as insulin resistance, increased adipogenesis, induction of gluconeogenesis, epigenetic mechanisms, chronic inflammatory state, and oxidative stress. Anti-obesity drugs such as Semaglutide and Tirzepatide are currently used in therapies for obese patients and exert remarkable anti-obesogenic effects, determining weight loss and inhibition of insulin resistance. The impairment of the adipogenesis process and the inhibition of the differentiation of human bone marrow mesenchymal stem cells into adipocytes should also be considered to improve the therapeutic strategies for obesity. Notably, the ability of several flavonoids to inhibit adipogenesis has been described. Flavonoids are the most abundant polyphenols in the human diet and exhibit a wide range of biological properties, including antioxidant and anti-inflammatory effects. Furthermore, many flavonoids can modulate the activity of enzymes involved in epigenetic mechanisms, which play a crucial role in obesity development. The purpose of this review is the identification of those flavonoids able to exert anti-adipogenic and anti-obesity effects in both in vitro and in vivo experimental models, with the aim of combining these natural molecules, as adjuvants, with anti-obesogenic drugs to develop innovative therapeutic approaches for the treatment of obesity pathology.

## 1. Introduction

**Figure 1:**
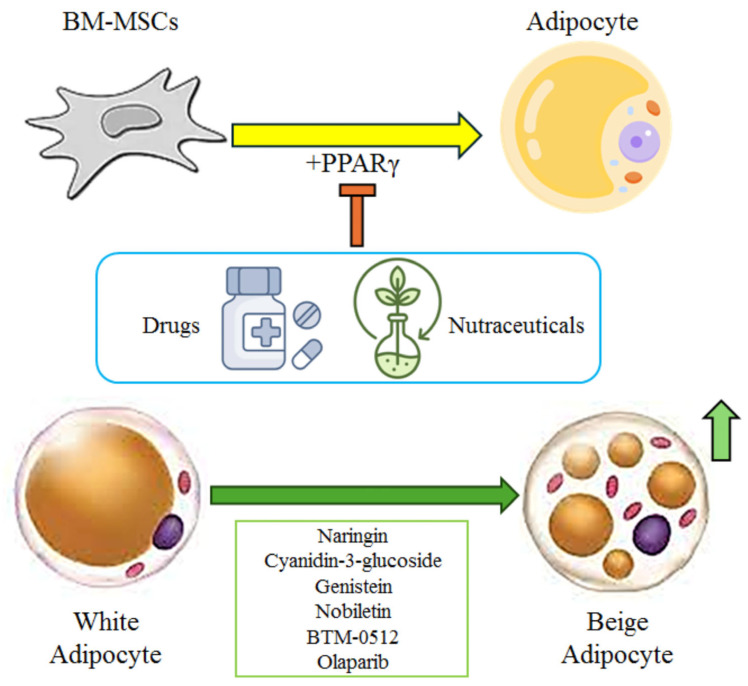
The combination of drugs and nutraceuticals can decrease the levels of PPARγ and, consequently, inhibit the differentiation of BM-MSCs into adipocytes. In addition, the flavonoids naringin, cyanidin-3-glucoside, genistein, and nobiletin and the drugs BTM-0512 and Olaparib can induce the brown adipocyte-like phenotype in white adipocytes, increasing the number of beige adipocytes. BM-MSCs: bone marrow mesenchymal stem cells; PPARγ: peroxisome proliferator activator protein-γ. The icons used for Figure 1 were downloaded from https://www.flaticon.com/ (accessed on 23 June 2025) [8].

Obesity is a chronic metabolic endocrine disease influenced by genetic, dietary, and behavioural factors involved in unbalanced energy metabolism that determine an increase in body fat accumulation and adipose tissue [1]. Notably, a Body Mass Index (BMI) > 30 is considered moderately obese, while a BMI > 35 is considered severely obese in patients [2]. Obesity can cause various clinical complications, such as hyperlipidemia, type 2 diabetes mellitus (T2DM), and cardiovascular diseases [3,4]. Of note, mammals possess two different types of adipose tissue: white adipose tissue, which stores triglycerides, and brown adipose tissue, which produces heat [5]. Recent findings have shown that beige adipocytes are an inducible form of brown adipocytes present in white adipose tissue and that this additional type of adipocyte possesses biochemical pathways similar to those used by brown adipocytes [6]. Notably, the promotion of the brown adipocyte-like phenotype in white adipocytes to increase the number of beige adipocytes has shown potential as an anti-obesity strategy in preclinical experimental models [7]. Some flavonoids (such as naringin, cyanidin-3-glucoside, genistein, and nobiletin) and drugs can increase the levels of beige adipocyte-specific markers both in vitro and in vivo (Figure 1).

Of note, it was shown that the flavonoid naringin was able to induce the brown adipocyte-like phenotype in white adipocytes in vitro by increasing the levels of the fat browning markers like uncoupling protein 1 (UCP1), PR domain-containing 16 (PRDM16), PPAR-γ coactivator 1-alpha (PGC-1α), and by upregulating the expression of the beige adipocyte-specific genes such as CBP/p300-interacting trans activator with Glu/Asp-rich carboxy-terminal domain 1 (*Cited1*), T-box transcription factor (*Tbx1*), and Transmembrane Protein 26 (*Tmem26*) [9]. Notably, the flavonoid cyanidin-3-glucoside induced in vitro phenotypic changes to white adipocytes, such as smaller lipid droplets, and increased the protein levels of the beige adipocyte markers UCP-1, Cited1, and Tbx1 [10]. In an in vitro experimental model, the flavonoid genistein reduced mRNA levels of markers indicative of white adipocytes and increased mRNA levels of the markers of brown/beige adipocytes, such as *UCP1* [11]. Furthermore, the flavonoid nobiletin treatment in 3T3-L1 white adipocytes elevated the expression levels of beige-specific genes, such as cell death activator (*Cidea*), *Tbx1*, and *Tmem26*, and induced phosphorylated adenosine monophosphate-activated protein kinase (p-AMPK), which plays a role in the molecular pathway p-AMPK/Sirtuin 1 (SIRT1)/PGC-1α that activates the fat browning process [12]. Notably, the SIRT1 activator BTM-0512 can induce the formation of beige adipocytes through the increase in SIRT1, UCP1, and PRDM16 levels mediated by the biomarker Ski-interacting protein (Skip) [13,14]. Of note, the Poly(ADP-ribose) Polymerase (PARP) inhibitor Olaparib was able to induce morphological changes through the formation of smaller lipid droplets in white adipocytes derived from human adipose-derived mesenchymal stem cells and to increase UCP1, p-AMPK, and SIRT1 levels, leading to the formation of beige adipocytes [15]. Although these in vitro and in vivo findings are promising, clinical trials will be needed to assess the potency of these natural and synthetic molecules to induce the brown adipocyte-like phenotype in white adipocytes in patients. Even if new therapeutic strategies determine a successful increase in the number of beige adipocytes in humans, it remains to be seen whether persistent weight loss will be achieved [7].

Adipocytes have also been recognized for their secretory function, releasing a range of molecules collectively known as adipocytokines. These molecules can be divided into two categories: the substances specifically expressed only in adipose tissue (such as leptin and adiponectin) and the substances that are not specifically expressed by adipocytes, such as tumour necrosis factor-α (TNF-α), interleukin-6 (IL-6), interleukin-8 (IL-8), interleukin-1β (IL-1β), and Monocyte Chemoattractant Protein-1 (MCP-1) [16]. Leptin is a key regulator of satiety that acts on the weight regulation centre of the hypothalamus, determining a decreased appetite and increased energy consumption, thereby inhibiting body weight gain [17]. Decreased sensitivity to leptin (leptin resistance) is thought to be one of the main causes of obesity [18]. On the contrary, the peptide hormone ghrelin is the hunger regulator, and it increases appetite and fat metabolism, thereby inducing obesity development [19]. Of note, adiponectin can regulate energy homeostasis, glucose, and fat metabolism in organisms [20]. This adipocytokine possesses anti-inflammatory and insulin-sensitizing properties, and it is downregulated in obesity, leading to insulin resistance and metabolic dysfunctions [21]. Notably, adiponectin activates peroxisome proliferator activator protein-α (PPAR-α), inducing fatty acid oxidation and decreasing triglyceride levels in muscles and in the liver, and it induces an increase in the activity of the lipoprotein lipase (LPL) [22]. In addition, this adipocytokine can decrease the levels of the phosphoenolpyruvate carboxykinase (PEPCK) enzyme and of the pro-inflammatory markers TNF-α, IL-6, and IL-8 [22]. Notably, the adiponectin receptor (AdipoR) agonist AdipoRon can activate the AMPK/adiponectin/PPAR-α signalling pathway in muscles and liver, thereby counteracting the detrimental effects of obesity [23]. In obese patients, elevated levels of TNF-α and decreased adiponectin levels contribute to chronic inflammation [24]. In addition, a TNF-α-mediated decrease in glucose transporter type 4 (GLUT4) induces insulin resistance, determining the development of T2DM [25]. It was reported that TNF-α can activate protein kinase C-α (PKC-α), which inhibits the insulin receptor (INSR)-mediated phosphorylation of insulin receptor substrate 1 (IRS-1), determining the inhibition of the phosphoinositide 3-kinase (PI3K)/phosphorylated protein kinase B (p-Akt)/GLUT4 signalling and, consequently, the induction of insulin resistance and the development of T2DM [26]. Notably, adiponectin increases insulin secretion by pancreatic β cells and enhances fat burning in the body, while the increased production of the pro-inflammatory marker TNF-α determines a decrease in adiponectin levels and promotes the increase in adipose tissue [27], highlighting the interconnected molecular pathways regulated by adipocytokines (Table 1).

Of note, the pro-adipogenic transcription factor peroxisome proliferator activator protein-γ (PPAR-γ) is highly expressed in the adipose tissue, and it exists in two isoforms: PPAR-γ1 and PPAR-γ2 [28]. This transcription factor plays an important role in the regulation of the molecular mechanisms of adipogenesis, adipocyte differentiation, and lipid metabolism [29]. Adipogenesis begins with the differentiation of bone marrow mesenchymal stem cells (BM-MSCs) into adipocytes, encompassing four stages: growth arrest, mitotic clonal expansion, early differentiation, and terminal differentiation [30]. The differentiation of BM-MSCs into adipocytes is regulated by several transcription factors such as CCAAT/enhancer binding protein alpha (C/EBPα), sterol regulatory element-binding protein 1 (SREBP1), and PPAR-γ [31], which modulate the expression levels of genes involved in lipid metabolism, including fatty acid synthase (*FAS*), *LPL*, *PEPCK*, and *GLUT4* [31]. It was shown that SREBP1 and PPAR-γ belong to the same molecular mechanism and are both activated by the cAMP response element-binding protein (CREB) and CREB-regulated transcription coactivator (CRTC), indicating that the molecular pathway CREB/SREBP1/PPAR-γ significantly contributes to the development of obesity [21]. Notably, it was reported that the complex CREB/CRTC2 can increase the activity of the transcription factor SREBP2 and consequently, the levels of the target genes of SREBP2: HMG-CoA reductase (*HMG-CoA-R*), Proprotein Convertase Subtilisin/Kexin type 9 (*PCSK9*), and LDL-Receptor (*LDL-R*), regulating the lipid metabolism [32]. Of note, PPAR-γ belongs to the nuclear hormone receptor family of ligand-activated transcription factors, which includes three members: PPAR-α, peroxisome proliferator activator protein-β (PPAR-β), and PPAR-γ [33]. It was shown that PPAR-α improves insulin resistance, increases fatty acid catabolism, and reduces inflammation [34]. PPAR-β decreases the transcriptional activity of the pro-inflammatory marker nuclear factor kappa-light-chain-enhancer of activated B cells (NF-kB), enhances the protein levels of INSR, and regulates lipid metabolism [34]. It was reported that many compounds, such as prostaglandins, fatty acids, and oxidized phospholipids, act as PPAR ligands, activating specific molecular pathways that lead to the differentiation of BM-MSCs into adipocytes [35]. On the contrary, a transcriptional coactivator with a PDZ-binding motif (TAZ) promotes osteoblast differentiation through activation of runt-related transcription factor 2 (RUNX2)-dependent genes and suppression of adipocyte differentiation via the repression of PPAR-γ [36]. It was shown that Wingless/Integrated (Wnt)/β-catenin signalling can inhibit adipocyte differentiation by blocking the expression of PPAR-γ and C/EBPα [37]. Notably, the serine/threonine protein kinase AMPK can decrease the expression levels of late adipogenic markers, such as FAS, by inhibiting the transcriptional activity of PPAR-γ and C/EBPα [38]. In addition, AMPK can phosphorylate HMG-CoA-R and inhibit its catalytic activity [33]. It was reported that AMPK activates catabolic pathways such as fatty acid oxidation and inhibits anabolic pathways such as the synthesis of fatty acids and their storage in adipocytes [39]. Mature adipocytes play a pivotal role in the storage of fat in the form of triglycerides, and the amount of these cells increases significantly in obese patients [31]. Recent evidence shows that epigenetic modifications, such as DNA methylation and histone acetylation/methylation, influence adipocyte differentiation, increasing or decreasing the expression levels of adipogenic genes [40]. These epigenetic signatures are also influenced by metabolic status, dietary habits, chronic inflammatory state, and oxidative stress, predisposing mesenchymal stem cells to pathological increased adipogenesis [40]. Elevated reactive oxygen species (ROS) in obesity leads to adipogenic dysfunction by determining oxidative damage to DNA, proteins, and phospholipids and by activating the pro-inflammatory transcription factor NF-kB [41]. Oxidative stress also leads to excessive activation of PPAR-γ, resulting in increased lipid storage and adipocyte hypertrophy [42]. It was reported that both intracellular and dietary antioxidants maintain the regulatory equilibrium of PPAR-γ through the reduction in ROS levels, thereby ensuring that the activity of this transcription factor remains within the optimal range that supports physiological adipocyte differentiation and triglyceride storage in the body [42]. Notably, by counteracting the pathological conditions of chronic inflammation and oxidative stress, antioxidant bioactives create a more resilient metabolic environment, inhibiting insulin resistance and metabolic dysfunctions in obesity and its associated cardiometabolic diseases [41]. It was shown that antioxidant molecules can increase the activity of the enzyme paraoxonase-1 (PON1), which hydrolyzes lipid peroxides in LDL- and HDL-cholesterol, reducing lipid peroxidation and supporting vascular health [41]. Dietary antioxidants, such as flavonoids, represent innovative therapeutic tools in the management of obesity, inhibiting both oxidative stress and the chronic inflammatory conditions that lead to obesity development [43]. Flavonoids, abundant in fruits, vegetables, and edible plants, with most of them occurring under the glycosidic form, are classified into different subgroups: anthocyanins, flavones, flavanones, isoflavones, neoflavonoids, flavonols, and flavanols [44,45]. Our review summarizes the anti-obesogenic effects of anti-obesity drugs such as Semaglutide and Tirzepatide and the anti-adipogenic properties of flavonoids, which play an important role in counteracting obesity pathological condition through modulation of specific and interconnected molecular markers and pathways in in vitro and in vivo models. Furthermore, our review highlights the clinical trials that have assessed the efficacy of anti-obesity drugs and flavonoids in obese patients. The purpose of our review is the identification of those flavonoids able to exert anti-adipogenic and anti-obesity effects in both in vitro and in vivo experimental models, with the aim of combining these natural molecules with anti-obesogenic drugs to develop innovative therapeutic approaches for the treatment of obesity pathology.

## 2. Epigenetic Mechanisms of Obesity

Recent evidence demonstrates that epigenetic regulatory mechanisms play a major role in the development and progression of obesity [46,47]. Epigenetics indicates a set of reversible and heritable changes in gene functions that occur without a change in DNA sequence [48]. Notably, several mechanisms are involved in epigenetic regulation, among which the most studied are DNA methylation, histone post-translational modifications (PTMs), microRNAs (miRNAs), and long non-coding RNAs (lncRNAs) [49]. DNA methylation refers to the addition of a methyl group (-CH_3_) to the 5′ site of the cytosine ring, mainly in the context of a CpG dinucleotide, catalyzed by enzymes named DNA methyltransferases (DNMTs). CpGs are clustered in regions named CpG islands, often located at gene promoters, whose methylation contributes to gene expression regulation. Silenced gene promoters are indeed hypermethylated, while expressed genes are normally hypomethylated [50]. In obese individuals, aberrant DNA methylation at the promoter regions of genes related to energy homeostasis and regulation of metabolic pathways, such as the gene that codifies for leptin, has been reported [51]. Furthermore, aberrant DNA methylation patterns have been described for the promoters of other genes involved in lipid and glucose metabolism: adiponectin (*ADIPOQ*), insulin receptor substrate 1 (*IRS1*), phosphatidylinositol 3-kinase regulatory subunit 1 (*PI3KR1*), *TNF*, and *IL-6* [52]. It was shown that hypomethylation of *ADIPOQ, IRS1*, and *PI3KR1* genes was associated with a decrease in BMI and the inhibition of insulin resistance, while the hypermethylation of *TNF* and *IL-6* genes was associated with a decrease in inflammatory markers and, consequently, with the inhibition of the molecular pathways of obesity [52].

Among histone PTMs, the most studied are methylation, acetylation, phosphorylation, and ubiquitination [53]. Histone modifications cooperate with DNA methylation, affecting the tightness of DNA packaging and the accessibility of transcription factors to the promoter of specific genes, modulating their expression [53]. Histone PTMs are regulated by several enzymes, the most studied being histone acetyltransferases (HATs), histone deacetylases (HDACs), histone methyl transferases (HMTs), and histone demethylases (HDMs) [53]. Histone methylation and acetylation play a role in the regulation of several genes involved in adipocyte differentiation (such as *C/EBPA*, *C/EBPB*, *aP2*, and *PPARG*), and the histone deacetylase sirtuins, such as SIRT1 and silent information regulator 6 (SIRT6), are known to play a pivotal role in the regulation of energy metabolism [46]. Of note, it was shown that increased acetylation of H3K9 and H3K18 at the *TNF* gene and decreased H4 acetylation at the *GLUT4* gene were correlated with a chronic inflammatory state, hyperglycaemia, and obesity development [52]. Notably, the enzymes involved in the regulation of histone PTMs have been proposed as molecular targets for innovative therapeutic strategies against obesity [46]. It was reported in both in vitro and in vivo experimental models that the drug valproic acid possesses HDAC inhibitory properties and can increase fatty acid oxidation, improve hepatic lipid metabolism, and decrease blood glucose levels [54], while the HDAC inhibitor givinostat reduced, in vitro and also in vivo, the levels of the pro-inflammatory markers NF-kB, interferon-γ (IFN-γ), IL-1β, and TNF-α [52]. Furthermore, givinostat (also indicated as ITF2357) inhibited in vitro insulin resistance and the apoptosis of pancreatic cells, counteracting the detrimental effects of insulin resistance mechanisms, which can lead to T2DM and obesity development, and, in addition, this drug normalized streptozotocin (STZ)-induced hyperglycemia conditions in vivo [55]. Of note, it was shown that the drugs hydralazine and procainamide possess DNMT inhibitory activities and are being investigated in clinical trials for treating T2DM and obesity [52].

### Flavonoids Regulate Epigenetic Mechanisms

**Figure 2:**
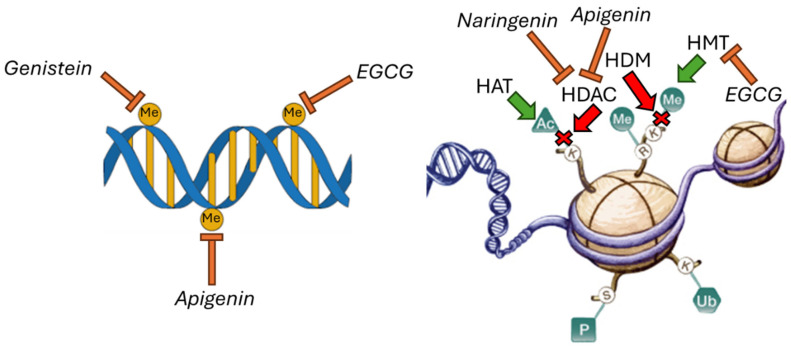
Flavonoids apigenin, genistein, epigallocatechin-3-gallate, and naringenin can modulate the epigenetic mechanisms of DNA methylation, histone deacetylation, and histone methylation. EGCG: epigallocatechin-3-gallate, HAT: histone acetyltransferase; HDAC: histone deacetylase; HMT: histone methyl transferase; HDM: histone demethylase; Me: Methylated; Ac: Acetylated; P: Phosphorylated; Ub: Ubiquitinated. Reproduced with permission. Copyright 2014, BioSocial Methods Collaborative, University of Michigan Institute for Social Research. The images used for Figure 2 were downloaded from https://biosocialmethods.isr.umich.edu/research-support/videos-tutorials/epigenetics-tutorial/ (accessed on 24 June 2025) [63].

It was reported that several flavonoids, such as kaempferol, genistein, apigenin, and quercetin, can influence epigenetic mechanisms, including DNA methylation and histone PTMs, miRNA levels, and chromatin remodelling, leading to adipogenesis reduction and an increase in fatty acid oxidation and, thus, offering an innovative therapeutic tool for obesity management [56,57]. Notably, the flavan-3-ol ester epigallocatechin gallate (EGCG) can inhibit the activity of DNMT and HMTs, determining a decrease in adipogenesis and an increase in both fatty acid oxidation and adiponectin levels in high-fat-diet-fed obese rats [58]; the isoflavone genistein was found to inhibit DNMT activity and modulate the expression of miRNAs involved in the inhibition of the adipogenesis process in 3T3-L1 murine preadipocytes [59]. Of note, it was shown that the flavonol apigenin can decrease DNMT and HDAC activity and levels, determining an increase in fatty acid oxidation, a decrease in the adipogenesis process, and the inhibition of chronic inflammation in high-fat-diet-fed C57BL/6J mice [60]. It was reported that the flavanone naringenin can inhibit adipogenesis and chronic inflammation and can induce fatty acid oxidation by regulating the expression of specific miRNAs involved in metabolic molecular pathways in diabetic C57BL/6J mice [61]. Notably, it was also reported that naringenin decreased HDAC1 levels in human HL60 cells, suggesting a role of this flavonoid in modulating histone acetylation [62]. The epigenetic mechanisms regulated by dietary flavonoids, such as genistein, EGCG, apigenin, and naringenin, are reported in Figure 2.

Of note, the evidence reported in the literature for the flavonoids EGCG, genistein, apigenin, and naringenin indicates that the anti-obesity effects of these phytochemicals are mediated by epigenetic mechanisms that regulate gene expression: DNA methylation, histone methylation, and histone deacetylation (Table 2).

## 3. Anti-Obesity Effects of Flavonoids: Biomarkers and Molecular Pathways

**Figure 3:**
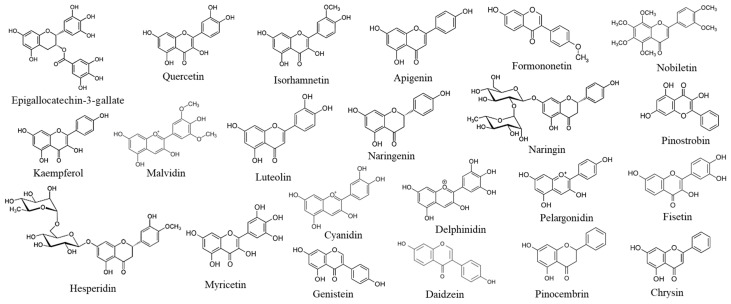
Chemical formulae of the flavonoids reported in this review.

The pathophysiology of obesity is highly complex and involves the activation or inhibition of many molecular pathways that contribute to its development. Flavonoids, like other phytochemicals, exhibit pleiotropic biological activities, as they are capable of simultaneously targeting multiple molecular mechanisms [64]. It was shown that several polyphenols and flavonoids possess anti-obesity properties [44]. Figure 3 shows the chemical formulae of flavonoids described in this study, which have been selected based on their well-documented anti-adipogenic and anti-obesity activities reported in [45].

These selected flavonoids belong to different subgroups—anthocyanins, flavones, flavanones, isoflavones, flavonols, and flavanols—as reported in Table 3.

**Figure 4:**
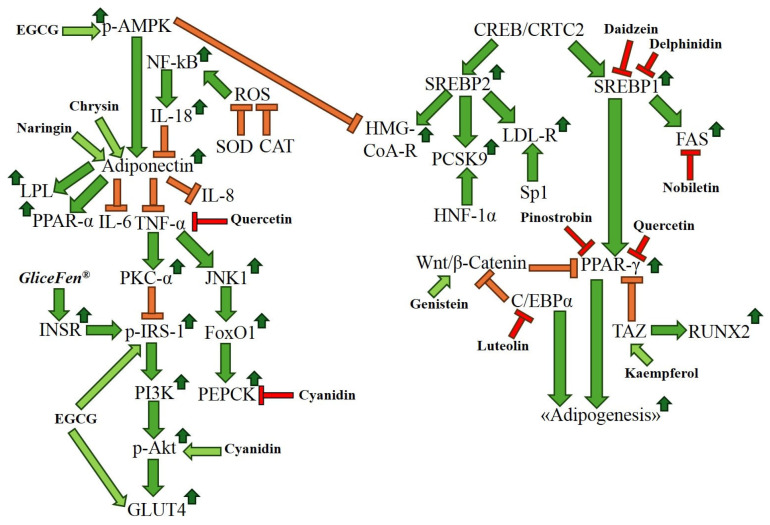
Molecular targets and molecular pathways that contribute to obesity pathology. The effects of representative flavonoids on the modulation of the levels of specific molecular markers involved in the adipogenesis process and in the development of obesity are reported. p-AMPK: phosphorylated adenosine monophosphate-activated protein kinase; NF-kB: nuclear factor kappa-light-chain-enhancer of activated B cells; IL-18: interleukin-18; ROS: radical oxygen species; CAT: catalase; SOD: superoxide dismutase; LPL: lipoprotein lipase; PPAR-α: peroxisome proliferator-activated receptor alpha; IL-6: interleukin-6; IL-8: interleukin-8; TNF-α: tumour necrosis factor alpha; JNK1: c-Jun N-terminal kinase 1; FoxO1: Forkhead box protein O1; PEPCK: phosphoenolpyruvate carboxykinase; PKC-α: protein kinase C alpha; p-IRS-1: phospho-insulin receptor substrate 1; INSR: insulin receptor; PI3K: phosphoinositide 3-kinase; *p*-Akt: phospho-Ak strain transforming; GLUT4: glucose transporter protein type-4; CREB: cAMP response element-binding protein; CRTC2: CREB-regulated transcription coactivator 2; SREBP1: sterol regulatory element-binding protein-1; SREBP2: sterol regulatory element-binding protein-2; FAS: fatty acid synthase; HMG-CoA-R: HMG-CoA-Reductase; LDL-R: LDL-Receptor; PCSK9: Proprotein Convertase Subtilisin/Kexin type 9; HNF-1α: hepatocyte nuclear factor-1 alpha; Sp1: specificity protein 1; PPAR-γ: peroxisome proliferator-activated receptor gamma; TAZ: transcriptional coactivator with PDZ-binding motif; RUNX2: runt-related transcription factor 2; C/EBP-α: CCAAT/enhancer binding protein alpha; Wnt/β-catenin: wingless-related integration site/beta-catenin; EGCG: epigallocatechin gallate.

Diets rich in bioactive compounds, such as flavonoids, are strongly associated with improvements in metabolic health, reducing rates of obesity and improving the lipid profile [43]. Flavonoids possess a wide range of beneficial biological activities, including antioxidant, anti-inflammatory, and anti-adipogenic biological properties, and can inhibit the molecular mechanisms that lead to obesity development [44,45]. Figure 4 describes key molecular pathways and biomarkers involved in the pathogenesis of obesity and the molecular targets of the adipogenesis process and obesity development that are modulated by several flavonoids.

It was shown that the flavonol fisetin can inhibit lipid accumulation and suppress early stages of adipocyte differentiation by inhibiting the expression of PPAR-γ in 3T3-L1 cells through the induction of SIRT1-mediated deacetylation of PPAR-γ, which leads to suppression of PPAR-γ transcriptional activity [65]. Furthermore, this flavonol inhibited the degradation of β-catenin, which is the biomarker that can induce the differentiation of BM-MSCs into osteoblasts, reducing the number of new adipocytes [64]. Of note, it was shown that the flavonoids pinocembrin and pinostrobin contained in the natural extract of *Myrceugenia euosma* (O.Berg) D.Legrand inhibited adipogenesis in 3T3-L1 cells by reducing the levels of PPAR-γ and SREBP1 [66]. Notably, the extract of *Carya cathayensis* Sarg. enriched in the flavanones pinocembrin and pinostrobin reduced total cholesterol, triglyceride, and free fatty acid levels in vivo [67]. It was reported that the pinocembrin treatment reduced body weight gain and the levels of PPAR-γ in an in vivo experimental model of obesity [68]. Another study showed that pinostrobin inhibited the differentiation into mature adipocytes of both mouse 3T3-L1 and human PCS-210-010 preadipocytes, and this flavonoid decreased the levels of the pro-adipogenic markers PPAR-γ and SREBP1 [69]. It was shown that chrysin exerted anti-obesity effects in vitro and in vivo by reducing the levels of PPAR-γ [70]. Notably, the flavone nobiletin inhibited the transcriptional activity of PPAR-γ and C/EBPα in an in vitro experimental model, demonstrating the anti-obesity effects of this citrus flavonoid [71]. Of note, the treatment with the isoflavone formononetin in 3T3-L1 cells reduced the levels of C/EBPα and PPAR-γ and increased the levels of β-catenin, leading to the inhibition of the adipogenesis process [72]. Another study showed that the anthocyanidin pelargonidin reduced the protein levels and the transcriptional activity of PPAR-γ in 3T3-L1 cells, indicating that this compound possesses anti-adipogenic properties in vitro [73]. Notably, in vitro results obtained in human mesenchymal stem cells (MSCs) indicate that the anthocyanidin malvidin induced the expression of RUNX2 and bone morphogenetic protein 2 (BMP-2), determining the differentiation of MSCs into osteoblasts and inhibiting the differentiation into adipocytes [74]. The same authors showed that delphinidin inhibited the MSC adipogenesis by downregulating fatty acid binding protein 4 (FABP4) [74]. Notably, the anthocyanidin delphinidin-3-O-β-glucoside in 3T3-L1 adipocytes downregulated the expression of PPAR-γ, SREBP1, and C/EBPα [75]. Another study showed that the flavonoid cyanidin can inhibit the adipogenesis process in 3T3-L1 adipocytes by downregulating the adipogenic markers PPAR-γ and C/EBPα [76]. It was reported that in an in vivo experimental model, the cyanidin-3-O-galactoside-enriched *Aronia melanocarpa* (Michx.) Elliott extract decreased serum levels of triglycerides, total cholesterol, and LDL-cholesterol, and reduced the expression levels of SREBP1 and PPAR-γ [77]. Of note, the flavonol myricetin exerted anti-obesity effects in 3T3-L1 adipocytes by decreasing the levels of PPAR-γ, C/EBPα, and SREBP1 [78]. In addition, it was shown that the extract of green citrus × junos Siebold ex Tanaka peel enriched in the flavanones naringin and hesperidin decreased, in vitro, the levels of the adipogenic markers C/EBPα, SREBP1, and PPAR-γ, while in vivo, this extract reduced body weight and serum lipid levels [79]. Notably, the treatment with the flavonol isorhamnetin decreased the expression levels of PPAR-γ in 3T3-L1 adipocytes, indicating anti-adipogenic properties of this flavonoid [80]. It was shown that the flavonol kaempferol decreased the levels of the adipogenic markers PPAR-γ, c/EBPα, and SREBP1 in 3T3-L1 cells [81]. Another study indicated that kaempferol can induce osteoblast differentiation of BM-MSCs in vitro by increasing both the TAZ-mediated activation of the transcription factor RUNX2 and the TAZ-mediated inhibition of the transcription factor PPAR-γ, resulting in diminished adipocyte differentiation [82]. Notably, it was reported that the extract of the plant *Eremochloa ophiuroides* (Munro) Hack., enriched in the flavone luteolin, exerted anti-adipogenic effects in 3T3-L1 adipocytes by decreasing the levels of C/EBPα and PPAR-γ [83]. Another study showed that a quercetin-enriched onion peel extract exerted anti-obesity effects in 3T3-L1 cells by decreasing the expression levels of key adipogenic genes *PPAR-γ* and *C/EBPα* [84]. Notably, quercetin treatment of OP9 mouse stromal cells remarkably decreased the levels of the adipogenic transcription factors C/EBPα, PPAR-γ, and SREBP1 [85]. It was shown that the isoflavones daidzein and genistein suppressed adipogenic differentiation of MSCs by inhibiting the expression of PPAR-γ and SREBP1 and activating the Wnt/β-catenin pathway [86]. Furthermore, daidzein treatment in obese rats decreased the expression levels of PPAR-γ, indicating an in vivo anti-obesity effect of this isoflavone [87]. Of note, it was reported in an in vitro experimental model that genistein suppressed key adipogenic markers, such as PPAR-γ and C/EBPα, and this phytochemical was more effective than its glycosylated form, genistin, in reducing lipid accumulation in the investigated cells [88]. It was shown that EGCG in 3T3-L1 cells exerted anti-obesity effects through the downregulation of PPAR-γ, SREBP1, and C/EBPα [89,90]. Furthermore, this flavonoid increased the levels of β-catenin and activated the Wnt/β-catenin pathway, leading to the inhibition of the molecular pathways of the adipogenesis process [89,90]. Of note, it was shown that apigenin exerted anti-obesity effects in vitro by reducing SREBP1 levels and in obese mice by decreasing the levels of total cholesterol and of the transcription factor PPAR-γ [91,92]. Notably, it was reported that naringenin treatment of 3T3-L1 adipocytes remarkably reduced the protein levels of Signal Transducer and Activator of Transcription 5A (Stat5A) and PPAR-γ, determining the inhibition of adipogenesis and the impairment of the function of mature fat cells [93]. Table 4 describes the molecular markers modulated by the flavonoids reported in this review to exert anti-adipogenic effects and to regulate epigenetic mechanisms involved in obesity development.

### 3.1. Anti-Diabesity Activities of Flavonoids

The concurrent occurrence of obesity and T2DM is indicated as the pathology diabesity [94]. Flavonoids possess a wide range of beneficial biological activities, including anti-adipogenic, anti-inflammatory, and antioxidant biological properties, and can inhibit the molecular mechanisms of insulin resistance that contribute to diabesity development [95]. It was shown that quercetin increased the levels of the antioxidant markers superoxide dismutase (SOD) and catalase (CAT), decreased the levels of the pro-inflammatory markers IL-6 and TNF-α, reduced both the body weight and blood glucose levels, and decreased the mRNA and protein levels of the biomarker secreted frizzled-related protein 4 (SFRP4) in an in vivo diabesity experimental model [96]. SFRP4 is a diabesity biomarker induced by an increase in IL-1β levels, and this inflammatory mediator hinders the exocytosis of insulin-secreting granules from the pancreatic β-cells, leading to T2DM development [96]. Of note, in the context of diabesity, the flavonoid EGCG can decrease body weight, adipose mass, cholesterol, and triglyceride levels and, in addition, can inhibit the mechanisms of insulin resistance, improve glucose metabolism, and increase the levels of p-AMPK, p-IRS-1, and GLUT4 in vivo to counteract the detrimental effects of T2DM [97]. Furthermore, it was shown that EGCG treatment can decrease the expression levels of genes involved in gluconeogenesis (such as *PEPCK*) and synthesis of fatty acids in an in vivo experimental model of diabesity, demonstrating both its anti-diabetic and anti-obesity biological properties [98]. Of note, it was reported that the cyanidin 3-glucoside treatment reduced blood glucose levels and improved insulin sensitivity in vivo [99]. The same study showed that the treatment with this flavonoid significantly decreased the levels of the pro-inflammatory markers TNF-α and MCP-1 and of the lipogenic markers FAS and SREBP1, induced an increase in p-Akt levels, and inhibited the molecular pathway TNF-α/c-Jun N-terminal kinase 1 (JNK1)/Forkhead box protein O1 (FoxO1)/PEPCK, hindering the activation of the gluconeogenesis process to counteract, in an in vivo experimental model, the detrimental effects of hyperglycemia [99]. Table 5 shows the markers upregulated or downregulated by the reported flavonoids for exerting anti-diabesity effects.

### 3.2. Anti-Obesity Effects of Flavonoids in Clinical Trials

A recent review has described several studies indicating that the anti-obesity, anti-inflammatory, and antioxidant properties of many flavonoids support the clinical use of flavonoid-based nutraceuticals to improve outcomes in patients affected by obesity and dyslipidemia [100]. Notably, results from a randomized, double-blind, placebo-controlled study have shown that a nutraceutical obtained from the leaves of *Cynara cardunculus* L. and containing chlorogenic acid and luteolin-7-glucoside significantly reduced the levels of total cholesterol, triglycerides, and LDL-cholesterol in patients with pre-obesity when administered at a dose of 150 mg/day for 6 months [101]. In addition, this nutraceutical intervention significantly decreased the body weight and the glycated hemoglobin (HbA1c) levels in the enrolled patients with pre-obesity [101]. Of note, it was reported in a double-blind, placebo-controlled, randomized clinical trial that the supplementation of naringenin to obese patients for 4 weeks significantly reduced BMI, visceral fat, and the levels of total cholesterol, triglycerides, and LDL-cholesterol [102]. Furthermore, the results of another clinical trial showed that 200 mg/day of naringenin for 4 weeks significantly increased serum levels of HDL-cholesterol and decreased the serum levels of total cholesterol, triglycerides, and LDL-cholesterol in the enrolled obese patients [103]. Notably, the results of a double-blind, randomized clinical trial conducted in a group of patients diagnosed with dyslipidemia showed that the administration of 450 mg naringin daily for 3 months significantly decreased BMI, total cholesterol, and LDL-cholesterol and, in addition, induced a significant increase in adiponectin levels (0.82 ± 0.25 μg/mL in the naringin-treated group vs. 0.59 ± 0.19 μg/mL in the placebo group), indicating the anti-obesity effects of naringin [104].

## 4. Anti-Obesity Drugs

**Figure 5:**
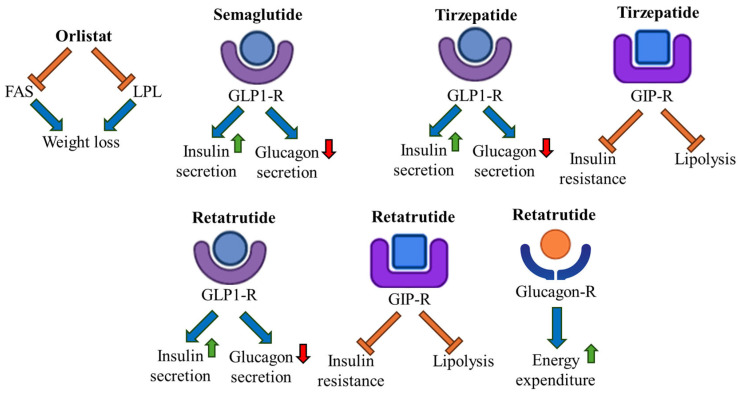
Molecular mechanisms regulated by anti-obesity drugs. The anti-obesity effects of the drugs Orlistat, Semaglutide, Tirzepatide, and Retatrutide, and the receptors with which they interact, are shown. FAS: fatty acid synthase; LPL: lipoprotein lipase; GLP1-R: GLP1 receptor; GIP-R: GIP receptor; Glucagon-R: glucagon receptor. The icons used for Figure 5 were downloaded from https://www.flaticon.com/ (accessed on 25 July 2025) [8].

Obesity, T2DM, and metabolic syndrome are currently the most prevalent metabolic disorders worldwide [105,106]. Treatments for obesity include dietary therapy, exercise, pharmacotherapy, and surgery. In clinical practice, primary prevention for obesity aims at avoiding the development of overweight and obesity conditions, while secondary prevention focuses on preventing the development of weight-related complications in obese patients [105]. Moreover, tertiary prevention aims at treating weight-related complications and preventing disease progression through pharmacotherapy [105]. Notably, the U.S. Food and Drug Administration approved several anti-obesity drugs, including Orlistat (which inhibits the enzyme FAS and the pancreatic lipases), Phentermine/Topiramate, Naltrexone/Bupropion, Liraglutide, Semaglutide, Setmelanotide, and Tirzepatide [107]. It was reported that the glucagon-like peptide-1 (GLP1) receptor agonist (GLP1-RA) Liraglutide can stimulate insulin release, inhibit glucagon secretion, slow gastric emptying, and increase the feeling of satiety after eating [108]. Of note, it was demonstrated that the peptide hormone GLP1 can increase glucose uptake and lipolysis and inhibit fat tissue inflammation in humans. In addition, GLP1 can stimulate insulin secretion and reduce glucagon secretion in the pancreas, leading to a reduction in blood glucose levels [105]. The drug Semaglutide can decrease hepatic gluconeogenesis, stimulate insulin release, and decrease glucagon secretion [105]. Of note, the synthetic cyclic peptide Setmelanotide can bind to the human melanocortin-4 receptor (MC4R) with high affinity, resulting in a decrease in appetite and a reduction of almost 10% in body weight [109]. The drug Tirzepatide is a dual glucose-dependent insulinotropic polypeptide (GIP) and glucagon-like peptide-1 receptor (GLP1-R) agonist used for the treatment of T2DM and obesity [110]. It was shown that the hormone GIP can stimulate insulin secretion, indicating the importance of the use of drugs that activate GIP/GLP-1 signalling [111]. The remarkable weight loss obtained after the administration of drugs such as Tirzepatide paved the way for the development of triple GIP/GLP-1/glucagon receptor agonists with the aim of obtaining synergistic metabolic effects [111]. Glucagon is a peptide hormone known to induce glycogenolysis and lipolysis, and to promote satiety and an increase in energy expenditure [112]. Retatrutide is an example of a drug that has an agonist effect on the GIP, GLP-1, and glucagon receptors. This triple receptor agonist has shown higher weight loss than surgical treatments and represents an improvement in the therapy for obese patients [112]. Notably, the drugs Cotadutide, Survodutide, Efinopegdutide, Pemvidutide, and Bamadutide have been developed as GLP1-R/glucagon receptor agonists and can increase energy expenditure and decrease food intake and hepatic lipogenesis, counteracting the detrimental effects of obesity [112]. Figure 5 shows the molecular mechanisms of obesity regulated by the drugs Orlistat, Semaglutide, Tirzepatide, and Retatrutide.

Of note, it was shown that, in high-fat-diet (HFD)-induced obese rats, the combination of the drug Orlistat and the flavonoid hesperidin decreased body weight, blood glucose levels, the Homeostatic Model Assessment of Insulin Resistance (HOMA-IR) index, and TNF-α levels [113]. Furthermore, the combination of Orlistat and hesperidin increased HDL-cholesterol values and reduced triglycerides, total cholesterol, and LDL-cholesterol levels [113]. Notably, the treatment based on the combination of hesperidin and Orlistat was more efficient than the treatment with Orlistat alone in increasing the HDL-cholesterol levels and in decreasing the values of LDL-cholesterol, TNF-α, and the HOMA-IR index, indicating that this flavonoid increased the anti-obesity effects of the drug Orlistat, improving both the glycemic and lipid profiles in vivo [113].

The co-occurrence of obesity and T2DM in the same patient is a pathological condition defined as “diabesity,” which is characterized by overlapping metabolic dysfunctions [114,115]. Insulin resistance and pancreatic β-cell deficiency are the primary causes of T2DM, while increased weight, chronic inflammation, hypertrophy, and hyperplasia of adipocytes are the primary causes of obesity [116]. Of note, it was shown that dysregulation of adipose tissue-derived adipocytokines and interleukins results in impaired insulin sensitivity in several organs, including the liver and pancreas [117]. In fact, it was shown that the pro-inflammatory cytokines IL-6, MCP-1, IL-1β, and TNF-α secreted by hypertrophic adipose tissue in obese individuals can disrupt insulin signalling and promote insulin resistance, contributing to the pathophysiology of diabesity [114]. Notably, standard pharmacological treatments for diabesity—such as metformin, sodium/glucose cotransporter 2 (SGLT-2) inhibitors, GLP1-RA, and dipeptidyl peptidase-IV (DPPIV) inhibitors—are effective in managing body weight and glycemic control, contributing to the inhibition of obesity [116,117]. Of note, it was shown that the combination of the GLP1-RA Liraglutide with the flavonoid quercetin in an in vivo diabesity experimental model significantly decreased the total cholesterol and triglyceride blood levels in the treated rats and, in addition, significantly reduced the HOMA-IR index, indicating that the combination of quercetin with the anti-obesity drug Liraglutide can inhibit the insulin resistance mechanisms that play a role in obesity development [118]. Furthermore, it was reported that the combination of Liraglutide and quercetin in diabetic rats significantly increased the protein levels of PI3K and IRS-1, restoring the insulin signalling pathway [119]. In addition, this treatment significantly decreased the triglyceride and total cholesterol blood levels in the diabetic rats, improving the lipid profile [119]. These studies showed that the combination of Liraglutide and quercetin was more effective than the individual treatments with quercetin or Liraglutide in improving both the glycemic and lipid profiles in vivo [118,119].

### Anti-Obesity Drugs and Flavonoid Combinations: A Novel Perspective

Researchers have reported the improved anti-obesity and anti-diabesity effects exerted in vivo by the combination of Orlistat with the flavonoid hesperidin and by the treatment based on the combination of Liraglutide with the flavonoid quercetin [113,118,119], but the molecular pathways and markers regulated by both the flavonoids described in this review and also by the anti-obesity drugs Orlistat, Liraglutide, Semaglutide, and Tirzepatide should be considered with the aim of identifying a shared signalling pathway and developing innovative therapeutic approaches targeting obesity. The drugs Semaglutide, Liraglutide, and Tirzepatide are GLP-1 RA, which activate the molecular pathway Protein Kinase A (PKA)/p-IRS/PI3K/p-Akt/GLUT4 to exert their therapeutic effects [120]. Of note, it was reported that the flavonoid EGCG can increase, in vivo, the levels of p-IRS-1 and GLUT4 and that the flavonoid cyanidin can increase, in an in vivo experimental model, the p-Akt levels, showing that these flavonoid activate the insulin signalling pathway to inhibit the insulin resistance mechanisms [97,98,99] and suggesting that these phytochemicals could be combined with Liraglutide, Semaglutide, and Tirzepatide to improve their efficacy. Since it was reported that the flavonoids chrysin, nobiletin, and naringin can increase the levels of adiponectin [104,121,122], which can block the TNF-α-mediated inhibition of the p-IRS-1/PI3K/p-Akt/Glut4 insulin signalling pathway (Figure 4), these flavonoids could be considered for the formulation of new nutraceuticals to be used in combination with anti-obesogenic drugs Liraglutide, Semaglutide, and Tirzepatide for developing innovative therapeutic approaches. Furthermore, the studies showing that the flavonoids nobiletin and cyanidin can decrease FAS levels in in vitro and in vivo models, respectively [12,99], and that the flavanone naringenin can decrease triglyceride levels and improve the lipid profile in clinical trials [102,103] suggest that these natural molecules could be combined with the drug Orlistat to improve its anti-obesity effects. On the other hand, it should be noted that new studies based on clinical trials will be needed to demonstrate, in humans, the improved therapeutic effects of a combination of anti-obesogenic drugs and selected flavonoids for counteracting the detrimental effects of obesity pathology.

## 5. Conclusions and Future Perspectives

This review outlined the therapeutic effects of several anti-obesity drugs and the anti-adipogenic activities of many flavonoids capable of inhibiting the differentiation of BM-MSCs into white adipocytes. Of note, several flavonoids are commonly used in the formulations of nutraceuticals because of their synergistic and pleiotropic biological properties [64,123]. However, a key limitation remains: the low bioavailability of many natural compounds used in nutraceutical formulations. This challenge can be addressed using innovative nano-delivery technologies, as extensively described in a recent review [64]. Notably, an example of a flavonoid-based nutraceutical is GliceFen^®^ (Mivell, Fano, Italy), which contains the flavanones naringenin and hesperetin, and the flavonol quercetin [124]. This formulation exerted anti-diabetic effects in vitro by decreasing the mRNA and protein levels of the pro-inflammatory marker Semaphorin 3E, decreasing the catalytic activity of caspase 1 and hyperglycemic enzyme DPPIV, and increasing the levels of INSR, determining the inhibition of the molecular mechanisms of insulin resistance, which can lead to obesity development [124]. The regulation of the biomarkers of the interconnected molecular pathways of insulin resistance, gluconeogenesis, adipogenesis, and chronic inflammation can contribute to the restoration of a healthy physiological condition, inhibiting the detrimental effects of obesity. Based on this evidence, future research should explore the therapeutic potential of flavonoid-enriched nutraceuticals as adjuvants in combination with conventional anti-diabetic and anti-obesity drugs. Such integrative and innovative therapeutic strategies may help to identify novel molecular and epigenetic biomarkers that play a pivotal role in the molecular mechanisms of obesity development and that can be modulated to improve the treatment of this chronic metabolic disorder.

## Figures and Tables

**Table 1:** Adipocytokines and their effects on energy homeostasis and obesity development.

Adipocytokines	Biological Activities	Pathologic Alterations in Obesity	References
Leptin	Increase in energy consumption; inhibition of body weight gain	Leptin deficiency and leptin resistance determine an increase in body weight	[17,18]
Adiponectin	Increase in insulin sensitivity; induction of fatty acid oxidation; decrease in triglyceride levels; inhibition of gluconeogenesis	Adiponectin decrease determines the development of insulin resistance and metabolic dysfunctions and the reduction in fatty acid oxidation	[20,21,22,23]
TNF-α	Induction of inflammation; induction of insulin resistance; induction of gluconeogenesis	TNF-α increase contributes to a chronic inflammatory state and to the development of metabolic dysfunctions	[16,25,26]
IL-6	Induction of inflammation	IL-6 increase contributes to a chronic inflammatory state	[16]
IL-8	Induction of inflammation	IL-8 increase contributes to a chronic inflammatory state	[16]
IL-1β	Induction of inflammation	IL-1β increase contributes to a chronic inflammatory state	[16]
MCP-1	Induction of inflammation	MCP-1 increase contributes to a chronic inflammatory state	[16]

**Table 2:** Flavonoids that modulate epigenetic mechanisms, which play a role in obesity development. The study type (in vitro and/or in vivo) is also indicated.

Flavonoids	Study Type	Epigenetic Regulations	References
EGCG	In vivo	Inhibition of DNA methylation; inhibition of histone methylation	[58]
Genistein	In vitro	Inhibition of DNA methylation; modulation of miRNA expression	[59]
Apigenin	In vivo	Inhibition of DNA methylation; inhibition of histone deacetylation	[60]
Naringenin	In vitro; in vivo	Modulation of miRNA expression; inhibition of histone deacetylation	[61,62]

**Table 3:** Classification of the flavonoids described in this study.

Flavonoids	Flavonoid Chemical Families	References
Pelargonidin	Anthocyanins	[43,44,45,46]
Malvidin	Anthocyanins	[43,44,45,46]
Delphinidin	Anthocyanins	[43,44,45,46]
Cyanidin	Anthocyanins	[43,44,45,46]
Chrysin	Flavones	[43,44,45,46]
Nobiletin	Flavones	[43,44,45,46]
Luteolin	Flavones	[43,44,45,46]
Apigenin	Flavones	[43,44,45,46]
Pinocembrin	Flavanones	[43,44,45,46]
Pinostrobin	Flavanones	[43,44,45,46]
Hesperidin	Flavanones	[43,44,45,46]
Naringin	Flavanones	[43,44,45,46]
Naringenin	Flavanones	[43,44,45,46]
Formononetin	Isoflavones	[43,44,45,46]
Daidzein	Isoflavones	[43,44,45,46]
Genistein	Isoflavones	[43,44,45,46]
Fisetin	Flavonols	[43,44,45,46]
Myricetin	Flavonols	[43,44,45,46]
Isorhamnetin	Flavonols	[43,44,45,46]
Kaempferol	Flavonols	[43,44,45,46]
Quercetin	Flavonols	[43,44,45,46]
EGCG	Flavanols	[43,44,45,46]

**Table 4:** Anti-adipogenic activities of flavonoids and their abilities to modulate epigenetic mechanisms described in this review. The in vitro/in vivo experimental models used for the studies, the molecular targets modulated by the reported flavonoids, and their administration mode are also indicated.

Flavonoids	Study Type	Administration Mode	Biological Activities	Outcomes of Studies	References
Fisetin	In vitro	Isolated flavonoid	Anti-adipogenic	Increase: β-catenin Decrease: PPAR-γ	[64,65]
Pinocembrin	In vitro; in vivo	Flavonoid-enriched extract; isolated flavonoid	Anti-adipogenic	Decrease: PPAR-γ, SREBP1	[66,68]
Pinostrobin	In vitro; in vivo	Flavonoid-enriched extract; isolated flavonoid	Anti-adipogenic	Decrease: PPAR-γ, SREBP1	[66,69]
Chrysin	In vitro; in vivo	Isolated flavonoid	Anti-adipogenic	Decrease: PPAR-γ	[70]
Nobiletin	In vitro	Isolated flavonoid	Anti-adipogenic	Decrease: PPAR-γ, C/EBPα	[71]
Formononetin	In vitro	Isolated flavonoid	Anti-adipogenic	Increase: β-catenin Decrease: C/EBPα, PPAR-γ	[72]
Pelargonidin	In vitro	Isolated flavonoid	Anti-adipogenic	Decrease: PPAR-γ	[73]
Malvidin	In vitro	Isolated flavonoid	Anti-adipogenic	Increase: RUNX-2, BMP-2	[74]
Delphinidin	In vitro	Isolated flavonoid	Anti-adipogenic	Decrease: FABP4, PPAR-γ, SREBP1, C/EBPα	[74,75]
Cyanidin	In vitro; in vivo	Flavonoid-enriched extract; isolated flavonoid	Anti-adipogenic	Decrease: PPAR-γ, C/EBPα, SREBP1	[76,77]
Myricetin	In vitro	Isolated flavonoid	Anti-adipogenic	Decrease: PPAR-γ, C/EBPα, SREBP1	[78]
Hesperidin	In vitro; in vivo	Flavonoid-enriched extract	Anti-adipogenic	Decrease: C/EBPα, SREBP1, PPAR-γ	[79]
Naringin	In vitro; in vivo	Flavonoid-enriched extract	Anti-adipogenic	Decrease: SREBP1, C/EBPα, PPAR-γ	[79]
Isorhamnetin	In vitro	Isolated flavonoid	Anti-adipogenic	Decrease: PPAR-γ	[80]
Kaempferol	In vitro	Isolated flavonoid	Anti-adipogenic	Increase: TAZ, RUNX2 Decrease: PPAR-γ, C/EBPα, SREBP1	[81,82]
Luteolin	In vitro	Flavonoid-enriched extract	Anti-adipogenic	Decrease: C/EBPα, PPAR-γ	[83]
Quercetin	In vitro	Flavonoid-enriched extract; isolated flavonoid	Anti-adipogenic	Decrease: PPAR-γ, C/EBPα, SREBP1	[84,85]
Daidzein	In vitro; in vivo	Isolated flavonoid	Anti-adipogenic	Increase: Wnt/β-catenin Decrease: PPAR-γ, SREBP1	[86,87]
Genistein	In vitro	Isolated flavonoid	Anti-adipogenic; regulation of epigenetics	Increase: Wnt/β-catenin Decrease: PPAR-γ, C/EBPα, SREBP1, DNMT	[59,86,88]
EGCG	In vitro	Isolated flavonoid	Anti-adipogenic; regulation of epigenetics	Increase: Wnt/β-catenin Decrease: PPAR-γ, SREBP1, C/EBPα, DNMT, HMT	[58,89,90]
Apigenin	In vitro; in vivo	Isolated flavonoid	Anti-adipogenic; regulation of epigenetics	Decrease: SREBP1, PPAR-γ, DNMT, HDAC	[60,91,92]
Naringenin	In vitro	Isolated flavonoid	Anti-adipogenic; regulation of epigenetics	Decrease: PPAR-γ, Stat5A, HDAC	[62,93]

**Table 5:** Biomarkers modulated by flavonoids to exert anti-diabesity effects.

Flavonoids	Study Type	Administration Mode	Outcomes of Studies	References
Quercetin	In vitro	Isolated flavonoid	Increase: SOD, CAT Decrease: IL-6, TNF-α, SFRP4	[96]
EGCG	In vivo	Isolated flavonoid	Increase: p-AMPK, p-IRS-1, GLUT4 Decrease: PEPCK	[97,98]
Cyanidin	In vivo	Isolated flavonoid	Increase: p-Akt Decrease: TNF-α, MCP-1, FAS, SREBP1, PEPCK	[99]

## Data Availability

No new data were created or analyzed in this study. Data sharing is not applicable in the review article.
